# Coexistence and Conflict between the Island Flying fox (*Pteropus hypomelanus*) and Humans on Tioman Island, Peninsular Malaysia

**DOI:** 10.1007/s10745-017-9905-6

**Published:** 2017-04-24

**Authors:** Sheema Abdul Aziz, Gopalasamy Reuben Clements, Xingli Giam, Pierre-Michel Forget, Ahimsa Campos-Arceiz

**Affiliations:** 1 Rimba, 22-3A Casa Kiara II, Jalan Kiara 5, 50480 Kuala Lumpur, Malaysia; 2 UMR 7179 CNRS-MNHN, Muséum National d’Histoire Naturelle, Département Adaptations du Vivant, 1 av du Petit Château, F-91800 Brunoy, France; 3 School of Environmental and Geographical Sciences, The University of Nottingham Malaysia Campus, Jalan Broga, Semenyih, 43500 Kajang, Selangor Malaysia; 4 Centre for Biological Sciences, Faculty of Natural and Environmental Sciences, University of Southampton, University Road, Southampton, SO17 1BG UK; 5 Kenyir Research Institute, Universiti Malaysia Terengganu, 21030 Kuala Terengganu, Terengganu Malaysia; 6 School of Aquatic and Fishery Sciences, University of Washington, Seattle, WA 98105 USA; 7 Department of Ecology and Evolutionary Biology, University of Tennessee, Knoxville, TN 37996 USA

**Keywords:** Conservation, Local communities, Human-wildlife conflict, Fruit bat, Pteropodidae, Tioman Island, Peninsular Malaysia

## Abstract

As tropical landscapes become increasingly human-dominated, conflicts between people and wildlife threaten ecological processes. Old World fruit bats such as flying foxes are especially susceptible to extinction risk because there is low interest in their conservation, particularly when they are considered pests. In order to arrest fruit bat declines, there is an urgent need to understand human-bat conflict and its implications. On a tropical island in Peninsular Malaysia, we conducted a questionnaire survey to investigate coexistence between people and the island flying fox (*Pteropus hypomelanus*). Among 119 respondents, knowledge of ecosystem services provided by flying foxes was extremely low. Most respondents held negative attitudes towards the bats, and older male locals were more likely to support killing them. This was also true for older owners of fruit trees who derived income from selling fruit, and experienced flying fox raids. Our results can be used to design appropriate interventions to support conservation efforts, and has important implications for managing conflicts between humans and synanthropic wildlife.

**Electronic supplementary material:**

The online version of this article (doi:10.1007/s10745-017-9905-6) contains supplementary material, which is available to authorized users.

## Introduction

Human-wildlife conflict is a major threat to ecological processes in tropical ecosystems. Many such ecological processes are sustained by Old World fruit bats (Chiroptera: Pteropodidae) found throughout the tropics and sub-tropics of Africa and Australasia (Marshall 1983). These bats play a crucial role in pollination and seed dispersal through their phytophagous diet, which in turn benefits human wellbeing (Fujita and Tuttle 1991; Kunz *et al.* 2011). However, despite these documented benefits, negative attitudes towards fruit bats persist amongst the general public (Pennisi *et al.* 2004; Thiriet 2010; Kingston 2016). Pteropodid bats, in particular flying foxes (*Pteropus* spp., *Acerodon* spp.), are frequently shot, persecuted, and even legally culled as pests of fruit crops (Bumrungsri *et al.* 2009; Epstein *et al.* 2009; Florens 2016). In addition, flying foxes have been hunted intensively for food and medicinal use (including in commercial trade), leading to severe declines throughout their range (Mildenstein *et al.* 2016). Estimates based on current deforestation rates predict that many fruit bat species in Southeast Asia may become globally extinct by the end of this century (Lane *et al.* 2006), with flying foxes being of particular concern due to intense hunting pressure. This has led to a widespread consensus that flying fox conservation and monitoring must be prioritised (Kingston 2010).

The situation is particularly urgent given that we still have a poor understanding of the implications of large-scale flying fox extinctions, especially on tropical islands. These bats are known to interact with plants over large spatial scales, performing ecological roles over vast transboundary areas (Epstein *et al.* 2009). They are likely to be important players in island ecosystems, where they often serve as principal pollinators and seed dispersers (Cox *et al.* 1991; McConkey and Drake 2015). Indeed, high flying fox densities are necessary for the maintenance of their ecological function as seed dispersers (McConkey *et al.* 2012). Yet local people remain unaware of the importance of flying foxes (e.g., Mahmood-ul-Hassan et al. 2011; Vincenot *et al.* 2015a; Weber *et al.* 2015), and this ignorance is compounded by cultural predilections for consuming flying fox meat (Wiles and Brooke 2009; Mildenstein *et al.* 2016).

Engagement of local communities, particularly those living in close proximity to flying foxes, is a crucial component of wider conservation actions needed to address this problem. Studies have shown that people’s attitudes towards wildlife are frequently influenced by factors such as age, gender, and culture (e.g., Kellert and Berry 1987; Dickman 2010; Koziarski *et al.* 2016), but in Southeast Asia we lack such social data needed to guide and support conservation action. In addition, while the issue of fruit crop raiding by bats has been extensively documented in some countries (e.g., Australia), the situation in Southeast Asia remains poorly understood (Aziz *et al.* 2016). Perceptions of zoonotic disease risk present further complications for pteropodid conservation (Demma *et al.* 2009; Thiriet 2010; Luby 2013).

We investigated the situation of coexistence between the island flying fox (*Pteropus hypomelanus*) and local people, as well as areas of potential conflict, on Tioman Island off the east coast of Peninsular Malaysia. Specifically, we aimed to identify: 1) whether people held positive or negative attitudes towards flying foxes; 2) reasons behind attitudes held; and 3) factors that might predict these attitudes. We gathered information from every household in one village on peoples’ knowledge, perceptions, experiences, and attitudes towards the flying foxes, and identified important socio-demographic factors that influenced these attitudes. Our findings can be used to better inform outreach and mitigation strategies aimed at reducing conflict between humans and synanthropic fruit bats in the tropics.

## Materials and Methods

### Study Site

**Fig. 1:**
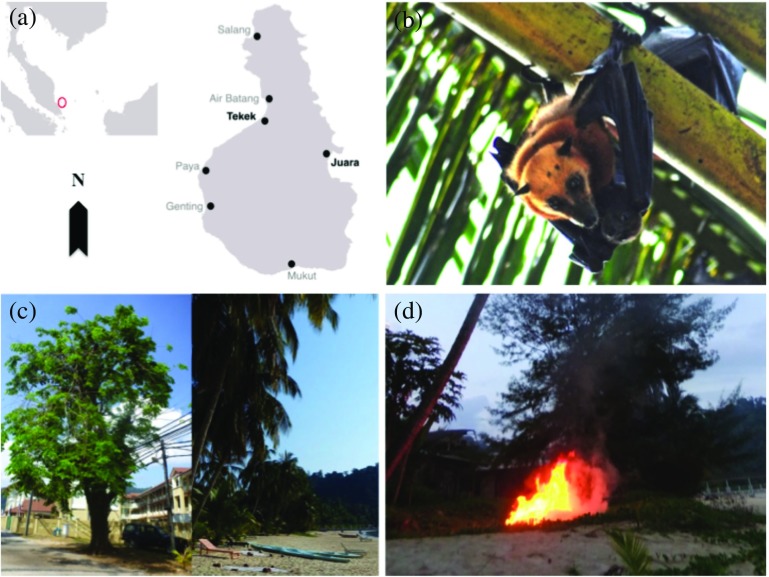
**a** Tioman Island; (**b**) *Pteropus hypomelanus*; (**c**) Flying fox roosts in Tekek (*left*) and Juara (*right*); (**d**) Fires used to smoke out flying foxes from roosts in Juara

Tioman Island (2°48′38″ N, 104°10′38″ E; 136 km^2^; Fig. 1a) is located 32 km off the east coast of Peninsular Malaysia in Pahang State. Much of the island is covered by lowland mixed dipterocarp forest and hill dipterocarp forest, an area designated as Pulau Tioman Wildlife Reserve (83 km^2^). The climate is uniformly warm and humid throughout the year (Hasan Basyri *et al.* 2001), with the northeast monsoon occurring from November to March (Bullock and Medway 1966). There are seven main villages on the island (Fig. 1a), totaling ~3400 people.

Around 60% of Malaysia’s population is classified as ethnic Malay, ~26% classified as ethnic Chinese, and the rest as ethnic Indian, indigenous, or ‘others,’ with the most widely professed religion being Islam, followed by Buddhism, Christianity, and Hinduism respectively (Department of Statistics Malaysia 2015; BBC 2016). On Tioman however, the population is predominantly ethnic Malay Muslims who do not hunt the bats for food or medicine due to religious dietary restrictions (e.g., http://islamic-laws.com/fooddrinks.htm). As the island’s marine area is also a designated Marine Park and a popular tourist destination, many local people are involved in the tourism industry (Abdul 1999).

Prior to this study, all villages on the island were visited during 4–7 April 2013. Informal conversations and interviews were conducted in each village as a basis for the study and these also provided useful insights and enhanced our understanding of the flying fox situation. We determined that flying foxes roost in only two locations on the island: the villages of Tekek and Juara, but that they regularly feed on cultivated fruit trees in all villages. Due to logistical, time, funding, and manpower constraints, the questionnaire survey was conducted only in Juara.

### Study Species

The island flying fox (*Pteropus hypomelanus*; also known as the variable or small flying fox; Fig. 1b) is the smallest flying fox species in Southeast Asia. It is listed as Least Concern on the IUCN Red List, although its population trend is decreasing (Francis *et al.* 2008). In Peninsular Malaysia it is confined mainly to small offshore islands, and is listed as Endangered on the Malaysian Red List (DWNP 2010).

On Tioman, the island flying fox roosts permanently in two villages: Tekek, the main and biggest village (~1260 people), located on the west coast, and Juara, the second largest village (~360 people) and the only one on the east coast (Fig. 1c). Monthly roost counts conducted during March–October 2015 yielded estimated ranges of 675–1033 individuals in Juara, and 2178–5385 individuals for the entire island.

Following Kingston (2010), we use the common term ‘flying fox’ to refer only to the genera *Pteropus* and *Acerodon*; only the former is present in Malaysia.

### Data Collection

We designed a questionnaire (Supplementary material 1) consisting of open-ended and fixed-response questions to obtain data on four main information groups: 1) socio-demographics; 2) knowledge and perceptions; 3) experiences; and 4) attitudes regarding flying foxes. The questionnaire was in formal Bahasa Malaysia (the standardised version of the Malay dialect officially used in Malaysia). A first draft was pilot-tested on three people (who were not subsequently included in the actual survey), and based on results and feedback it was amended and refined. To avoid possible bias resulting from the ordering of questions and sections, we included questions on positive, negative, and neutral aspects of flying foxes.

The questionnaire survey was carried out in Juara during March 2014–March 2015, generally during the last week of each month, excluding the monsoon period (Oct-Feb). The locations of households were first mapped with the help of locals to maximise the likelihood of interviewing every household. A household was defined as a group of people living collectively under one roof, and therefore included foreign owners and/or operators of resorts based in the village.

The questionnaire was administered by five female and two male enumerators via face-to-face interviews conducted in standard colloquial Malay. Enumerators targeted at least one female and one male from each household, preferably the heads of the household, with a minimum age limit of 18 years old. If either female or male household head was unavailable, another member of the required gender from the same household was surveyed. Each question was read aloud by the interviewer to the respondent, and the respondent’s answer was then recorded by the interviewer directly in the questionnaire. Where a question required ‘yes,’ ‘no,’ or ‘don’t know/not sure’ answers it was asked without providing answer options. As an added safeguard, these questions were followed up with an open-ended question (‘why?’ or ‘how do you know?’) to elicit further information and assess the reasoning behind the answer.

Our surveys conform to the research ethics criteria stipulated by The University of Nottingham Malaysia Campus. Consent from the village head was obtained prior to the questionnaire survey, after the general aims of the project and survey were first explained to him. Free, prior and informed consent (FPIC) to participate was also obtained from each respondent before commencing each interview. In order to maintain privacy, respondents’ identities and house locations were not recorded.

### Data Analyses

**Fig. 2:**
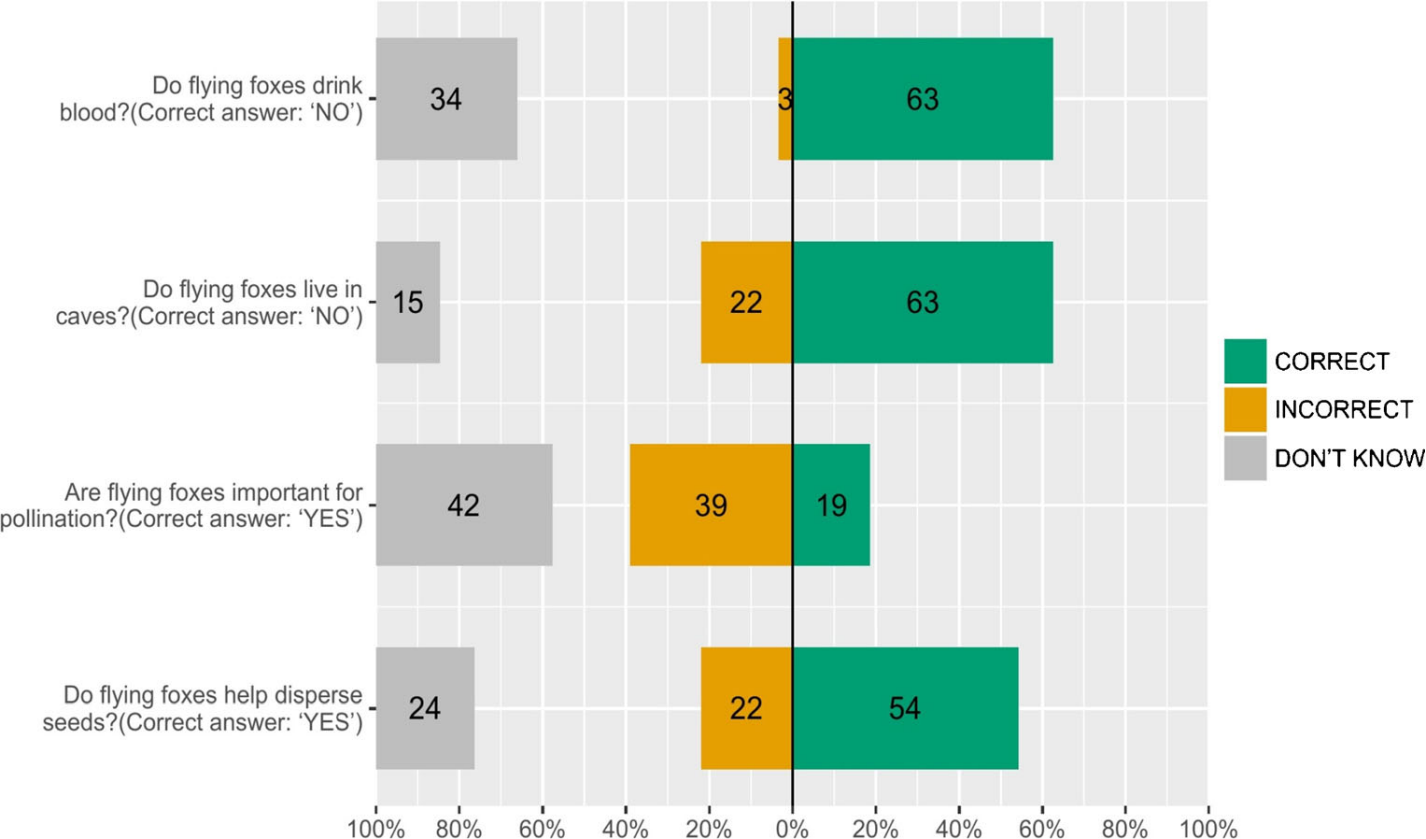
Knowledge of flying foxes amongst 119 respondents in Juara, Tioman

To characterise the attitudes of Juara’s community towards flying foxes, we asked four main questions (Fig. 2): whether they liked flying foxes [LIK], whether flying foxes should be conserved [CON], whether it would be good if flying foxes go extinct [EXT], and whether flying foxes should be killed [KIL].

In order to examine which combinations of six socio-demographic covariates (age [AGE], gender [GEN], local to Juara village [LOC], possession of formal education [EDU], possession of income [INC], ownership of fruit trees [OWN]) were most important in influencing peoples’ attitudes towards flying foxes, we constructed Generalised Linear Mixed-Effect Models (GLMMs; see Supplementray material 2 for R code) using an all-subsets multimodel inference framework (Burnham and Anderson 2003; Giam and Olden 2016). We used binomial (logit-link) GLMMs to model the binary (yes vs. no) response variables. Before running the GLMMs, we first assessed whether one or more pairs of candidate socio-demographic covariates were highly correlated (|coefficient values| > 0.5) in order to obtain more stable and interpretable parameter estimates. For binary vs. continuous covariates, we ran point bi-serial correlations and looked at Pearson coefficients. For binary vs. binary covariates, we looked at phi coefficients. To account for possible non-independence of answers due to different enumerators (ENU; 5 people) and different months during which questionnaires were administered (MON; 5 months), we allowed model intercepts to vary across both these random effects individually and in combination. However, we could not fit our models when MON was included and thus only included ENU as a random effect to prevent model over-parameterisation. We used sample-size corrected Akaike Information Criterion (AICc) to determine the best candidate model, Akaike weights (wAICc) to quantify the probability by which a given model is the best within the candidate models set, and the sum of Akaike weights (SW) to estimate relative variable importance (Burnham and Anderson 2003; Giam and Olden 2016). We calculated *R* ^*2*^_*m*_ to quantify the variance in the response variable that is explained by fixed effects in each GLMM (Nakagawa and Schielzeth 2013).

Among fruit tree owners, we constructed additional GLMMs to examine whether important socio-demographic covariates affecting the wider community’s attitudes would remain important when two covariates unique to fruit tree owners were factored in (selling their fruit [SEL] and having experience with fruit raiding [RAI]). All socio-demographic covariates were binary except AGE, whose values were standardised (i.e., values scaled to mean = 0, SD = 1) prior to running the models. In order to obtain binary response variables, ‘No’ and ‘Not sure’/‘Don’t know’ responses were pooled together and coded as ‘0’, and ‘Yes’ responses were coded as ‘1’. All analyses were conducted in R statistical environment 3.2.2 (R Development Core Team 2015).

## Results

From the 74 households, we obtained responses from 119 people (62 females, 57 males; Supplementary material 3). The median age of the respondents was 43 years (range: 18–76), with 70% identifying as being local to the village. Most (94%) of the respondents had received some formal education (i.e., school or university), with 45% receiving an education beyond primary level (i.e., >12 years old). An overwhelming majority of respondents identified as being ethnically Malay, Muslim in religious affiliation (95%), and practising the Malay culture (94%). A substantial majority of respondents (75%) owned fruit trees, but only 32% of fruit tree owners actually sold their fruit for income. None of the fruit tree owners relied on selling fruit as their main income, and appeared to do it opportunistically for side income.

### Knowledge and Perceptions

Ninety-four percent of respondents recognised the photo of a flying fox that was shown to them, with 10% of respondents identifying it generally as a bat (*kelawar* in Bahasa Malaysia), and 84% identifying it specifically as a flying fox (*keluang* in Bahasa Malaysia).

**Fig. 3:**
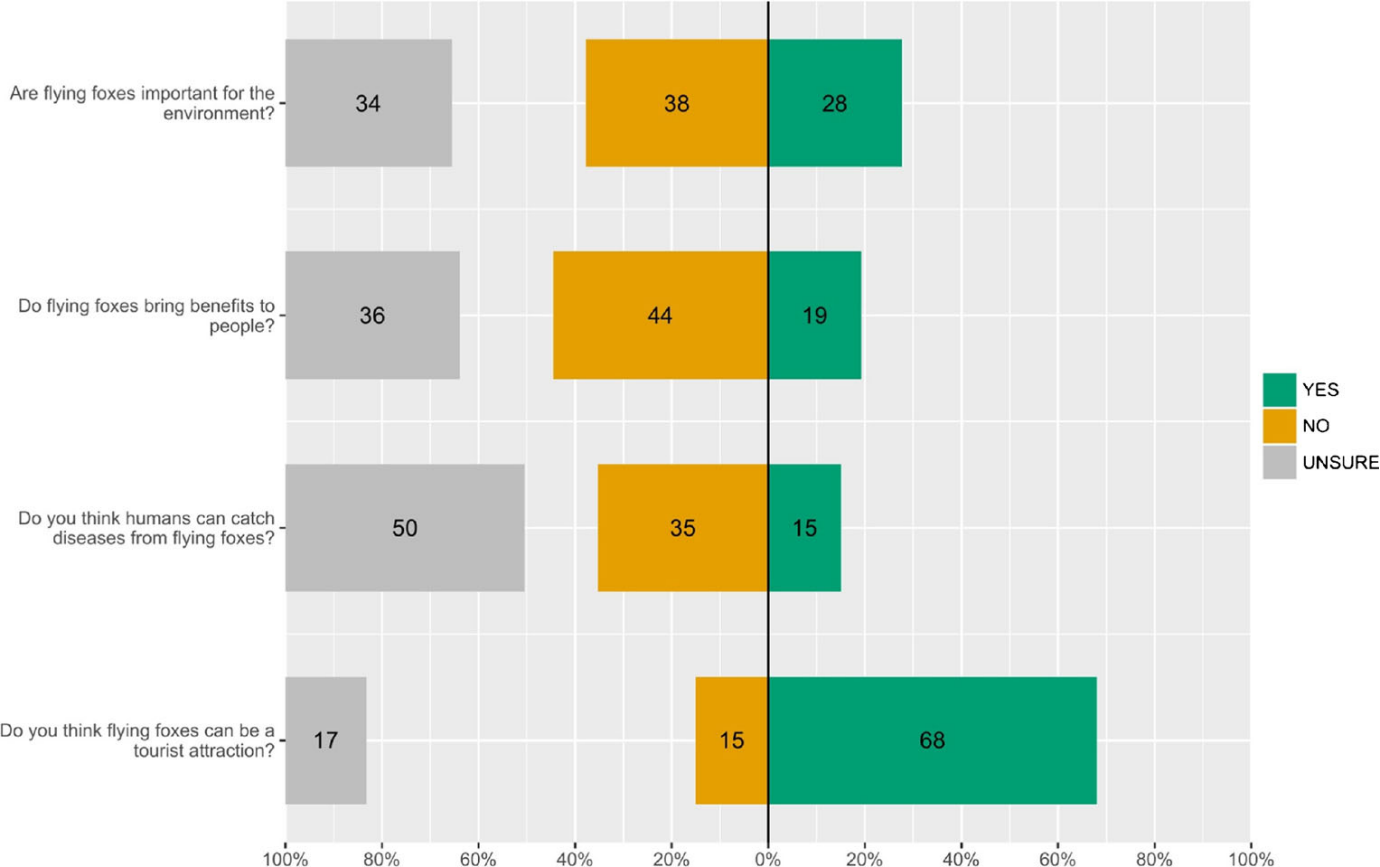
Perceptions of flying foxes amongst 119 respondents in Juara, Tioman

Only 9% of respondents were able to correctly answer all four questions devised to test accurate knowledge of flying foxes. Three percent of respondents said that flying foxes drink blood, and 22% said that flying foxes live in caves (Fig. 2). Only 13% of respondents agreed that flying foxes are important for pollination, compared to 55% who were aware that flying foxes help disperse seeds. However, awareness of ecological function did not necessarily translate to awareness of ecosystem services (Fig. 3), as only 28% of respondents stated that flying foxes were important for the environment, and only 19% stated that flying foxes brought benefits to people (however, eight respondents attributed health benefits to consumption of flying foxes). Thirty-six percent of respondents believed that flying foxes could be used as medicine, mostly for asthma (often also identified as a benefit), and when asked, “How do you know?” the most common answer was “This is what Chinese people say.”

Sixty-eight percent of respondents felt that flying foxes can be a tourist attraction. The majority of respondents did not perceive flying foxes as presenting any health threat, with only 21% stating that flying foxes harboured viruses, and only 15% stating that flying foxes could transmit diseases to humans.

### Experiences

Sixty-four percent of respondents said that flying foxes caused problems in the village, due to three main reasons: noise (12%), mess/smell from faeces (14%), and fruit raiding (24%). Sixty percent of fruit tree owners reported that flying foxes raided their fruit trees, although most also listed other animals as well, the most common being long-tailed macaques (*Macaca fascicularis*), black giant squirrels (*Ratufa bicolor*), red giant flying squirrels (*Petaurista petaurista*), and Sunda colugos (*Galeopterus variegatus*).

Most respondents (95%) said they did not kill the flying foxes; some explained that this was a “sensitive” issue due to Western tourists getting upset, and that the Department of Wildlife and National Parks had warned against killing flying foxes, which are a protected species. The most common method used (23% of respondents) to remove the bats was to light fires under trees to smoke them out (Fig. 1d); however, many respondents admitted that this was ineffective as the bats always return after a short time. Only 5% of respondents stated that they have shot the bats. A majority (56%) said they did nothing to chase the bats away.

Thirty percent of respondents reported that people have come to the village to eat, hunt, or buy flying foxes, with the most common answer identifying “Chinese people from elsewhere.” However, most also clarified that this happened 20–30 years ago, and no longer takes place.

### Attitudes

**Fig. 4:**
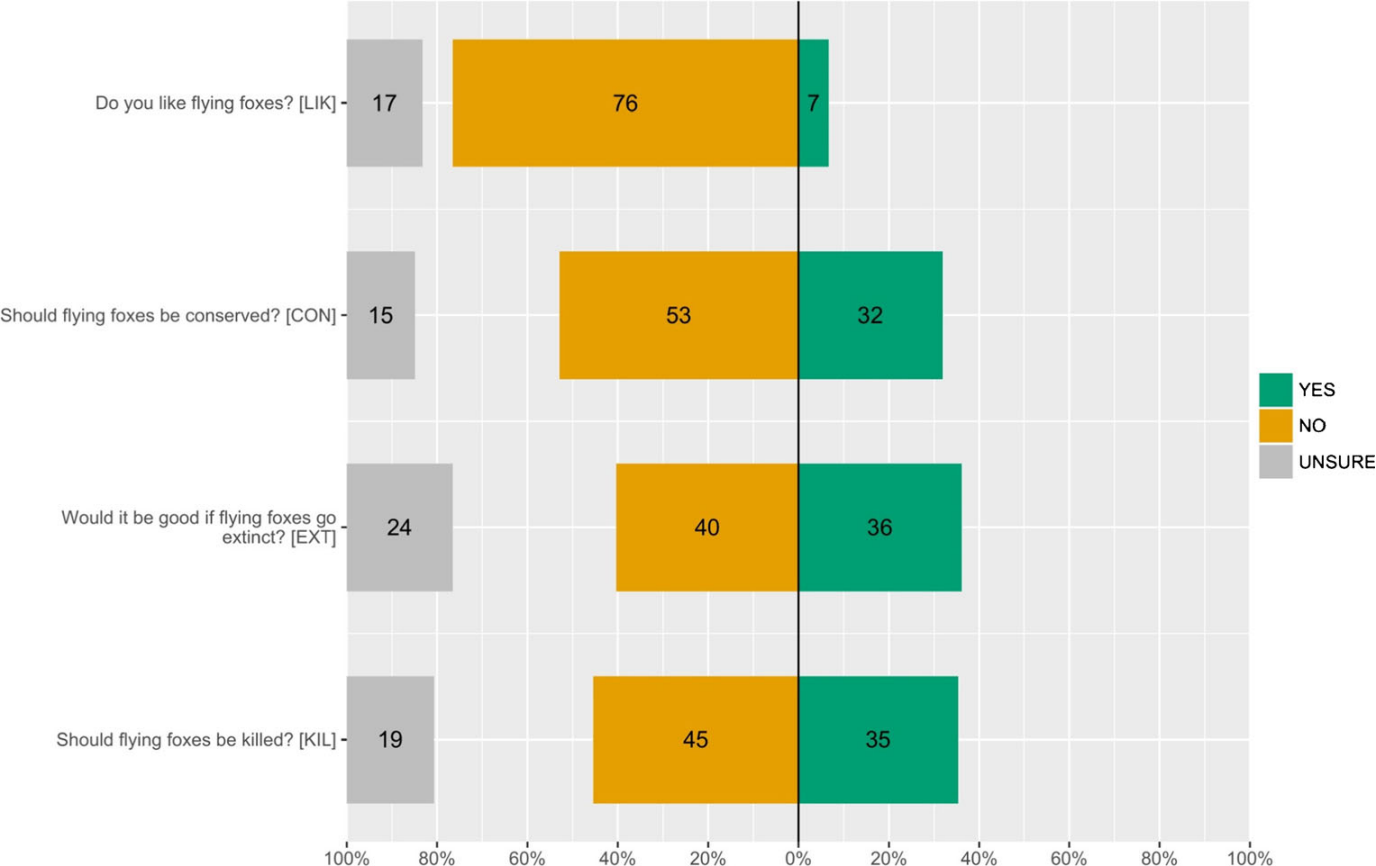
Attitudes of 119 respondents in Juara, Tioman towards flying foxes

Seventy-nine percent of 109 respondents (10 excluded due to unanswered questions) stated that they did not like flying foxes, with 39% saying that they should be killed (Fig. 4). Thirty-eight percent agreed it would be good if flying foxes went extinct, whereas only 32% said that they should be conserved.

A minority of respondents (25%) felt that flying foxes should be hunted specifically for food and medicine, with some explaining that this should be permitted for traditional Chinese medicine. However, respondents were evenly split in their response to a hypothetical total hunting ban, with a slightly higher percentage (46 v. 40%) supporting it. Reasons given for support expressed tolerance for coexisting with flying foxes, and were frequently moralistic invoking the need to avoid extinction, and the bats’ rights to live unharmed. Reasons for opposition cited flying foxes as a nuisance and the need for eradication or at least reduction in numbers.

### Socio-Demographic Correlates of Attitudes

As responses for [KIL] and [EXT] were correlated, we only investigated factors affecting [KIL], as the word ‘kill’ may convey a clearer meaning than ‘extinct.’ Across the entire Juara community, older [AGE] male [GEN] locals [LOC] were likely to want flying foxes killed [KIL] (wAICc =0.17, *R* ^*2*^_*m*_ = 0.31; Table 1A). In terms of relative variable importance assessed by sum of wAIC (SW), [AGE] was the most important factor (SW = 1.00), followed by [LOC] (SW = 0.82) and [GEN] (SW = 0.54). GLMMs could not be constructed for [LIK] as responses were skewed, and results were not reported for [CON] due to poor goodness-of-fit (marginal R^2^ values 0.14–0.16).

**Table 1:** The top three generalised linear mixed-effect models (GLMM) that relate attitudes [KIL] of: (A) the entire Juara community to socio-demographic predictors (age [AGE], gender [GEN], local to Juara village [LOC], having possession of education [EDU], possession of income [INC], ownership of fruit trees [FRU]; and (B) fruit tree owners to important socio-demographic predictors (identified from Part A but excluding [LOC] since they are all local) and two additional covariates (having experience with fruit raiding [RAI] and selling their fruit [SEL]). Enumerators [*ENU*] were coded as a random effect

Model	*k*	LL	AIC_*c*_	dAIC_*c*_	*w*AIC_*c*_	*R*_*m*_^*2*^
(A)						
KIL ~ AGE + GEN + LOC+(1|*ENU*)	5	-61.24	133.00	0.00	0.17	0.31
KIL ~ AGE + LOC+(1|*ENU*)	4	-62.74	133.80	0.82	0.11	0.27
KIL ~ AGE + INC + LOC+(1|*ENU*)	5	-62.07	134.70	1.62	0.07	0.29
(B)						
KIL ~ AGE + RAI + SEL+(1|*ENU*)	5	-39.14	89.10	0.00	0.18	0.38
KIL ~ AGE + GEN + RAI+(1|*ENU*)	5	-39.22	89.3	0.15	0.16	0.37
KIL ~ AGE + RAI+(1|*ENU*)	4	-40.40	89.40	0.24	0.16	0.34

Term abbreviations are defined as follows: *k* = number of parameters, LL = maximum log-likelihood, dAIC_*c*_ = difference in AIC_*c*_ for each model from the most parsimonious model, *w*AIC_*c*_ = AIC_c_ weight, and *R*_*m*_^*2*^ = marginal R^2^ according to Nakagawa and Schielzeth 2013

Among fruit tree owners, additional factors influenced attitudes; older people [AGE] who sold their fruit [SEL] and experienced fruit tree raiding [RAI] were most likely to want flying foxes killed (wAICc =0.22, *R* ^*2*^_*m*_ = 0.34; Table 1B). [RAI] was the most important factor (SW = 0.93), followed by [AGE] (SW = 0.89) and [SEL] (SW = 0.48).

## Discussion

Our study provides novel insights into coexistence and conflict involving flying foxes and a native Malay-Muslim population inhabiting a tropical island in Southeast Asia. It also shows that while a situation may appear peaceful to casual visitors, negative attitudes can exist and need to be addressed (e.g., Vincenot *et al.* 2015a).

Our results mirror similar situations in Australia where flying foxes also roost amongst humans (Larsen *et al.* 2002; Tait *et al.* 2014; Kung *et al.* 2015), where local residents express discomfort at the proximity of flying foxes to their homes and an unwillingness to share their living space with the bats (ABS 2016; Snowdon 2016), resulting in negative media coverage (e.g., Bennion 2016; De Lore 2016; Owen 2016; Snowdon 2016). These situations appear common when increased availability of food resources in human-dominated areas attract flying foxes (e.g., Parry-Jones and Augee 2001; Williams *et al.* 2006; Welbergen and Eby 2016). This certainly seems to be the case in Tioman despite the proximity of largely intact primary rainforest containing a diverse range of wild food plants (Latiff *et al*. 1999), which supports the argument that this is mostly a behavioural response and not driven by deforestation (Tait *et al.* 2014). In addition, we also believe that the similarities between Tioman and Australian case studies demonstrate how flying foxes may readily establish in human settlements where people do not hunt them.

Comparing the situations in Juara and Australia, slightly different reasons were given for people’s negative attitudes. Although noise and smell were commonly cited, the community in Juara also mentioned fruit raiding as a major concern, which was not a complaint amongst Australian residents whose more pressing concerns included disease transmission, damage to property, and impact on water quality (Larsen *et al.* 2002; Tait *et al.* 2014; Kung *et al.* 2015). The low concern regarding disease transmission in Juara is likely due to a lack of awareness (Fig. 4). However, whilst fruit raiding is a common complaint amongst commercial fruit farmers regardless of country (Mahmood-ul-Hassan *et al.* 2011; Aziz *et al.* 2016), the community in Juara does not depend on fruit farming for their main income. The few people who do sell their fruit (22% of respondents) did so only occasionally. Our results suggest that economic considerations are not the main driver of attitudes within the wider community in Juara. This is similar to Koziarski *et al.* (2016) who found that economic considerations were not as influential as education, psychological, and demographic attributes in wildlife conflict perceptions. Despite this, 39% of fruit tree owners in Juara wanted flying foxes killed. Cousins and Compton (2005) also found that fruit crop raiding was the most common reason for negative local attitudes towards flying foxes on the Cook Islands. This contrasts with qualitative research in central Myanmar, which suggests positive local attitudes and no evidence of conflict between flying foxes and villagers despite bats feeding on fruit trees also utilised by people (Win and Mya 2015).

It is unclear to what extent culture might play a role in such situations. For example, a study in Kerala, India, recorded positive community attitudes towards fruit bats including flying foxes (Deshpande and Kelkar 2015). In addition, there was high awareness of seed dispersal services by bats, which local people involved in agroforestry practices acknowledged as being beneficial. To date, that is the only study to show a link between awareness of ecosystem services and positive attitudes towards flying foxes. However, it is unknown whether the respondents in this study had fruit bats roosting amongst them, as shared living space is also one major reason for conflict. Notably, in one Australian study (Kung *et al.* 2015), 72% of respondents thought that the ecological role of flying foxes was important, yet 57% still felt flying foxes were a cause for concern in their community. Cultural attitudes towards coexisting with wildlife varies across different communities; e.g., religious and socio-cultural factors in Sri Lanka have led to historically positive and tolerant attitudes towards wildlife (Fernando *et al.* 2005), and this may be more prevalent in South Asia compared to Southeast Asia.

Nevertheless, awareness of ecosystem services could still potentially improve a community’s perceptions towards flying foxes. In Juara the community had very low awareness of ecosystem services provided by flying foxes (Fig. 3); indeed, only four respondents stated specifically that flying foxes help pollination. Although a higher percentage of respondents were aware that flying foxes dispersed seeds – probably due to this activity being more visible – they did not necessarily make the link to this as beneficial to people in any way. Knowledge of ecological function did not necessarily translate to awareness of ecosystem services, as very few stated that flying foxes brought benefits to people (Fig. 4; however, eight respondents attributed ‘health benefits [to] consuming flying foxes’!). Unlike Reid (2016), we were unable to investigate effects of knowledge on attitudes as it was impossible to separate this effect from perceptions and experiences. Depending on type and amount of knowledge (obtained through experiences), two people could have differing perceptions of an ecological function; e.g., seed dispersal might be perceived positively if associated with ecosystem balance, but perceived negatively if associated with fruit raiding, mess, and noise. Koziarski *et al.* (2016) point out that knowledge (derived through education) can have mixed effects, and in some cases can even increase fear and negative attitudes towards wildlife.

In addition to problems caused by flying foxes, low awareness of ecosystem services may partly explain why the majority of respondents (79%) in our study did not like flying foxes, and also why some (38%) felt it would be good for flying foxes to become extinct (Fig. 2). When a species is not perceived as having any kind of value to people, the disappearance of that species will not be seen as a loss. Similar situations have been reported from Thailand (Weber *et al.* 2015), Pakistan (Mahmood-ul-Hassan *et al.* 2011), and Japan (Vincenot *et al.* 2015b), where all or most people were unaware of ecosystem services provided by fruit bats, and perceptions of flying foxes as orchard pests causing economic loss often result in bat fatalities. The positive example from India (Deshpande and Kelkar 2015) suggests that when people are more aware, they might be willing to overlook negative experiences if these are outweighed by the overall beneficial aspects of the animals. However, we cannot rule out the possibility that a strong cultural background of tolerance could also be an influencing factor (Dickman 2010).

Among our respondents, older male locals tend to feel more negatively towards flying foxes, and this likely increases with the person’s length of residence. Koziarski *et al.* (2016) reported similar results from human-carnivore conflict in Tanzania. Cumulative personal encounters, combined with misconceptions and limited knowledge about the animals (Fig. 3) can play a role in shaping attitudes (Kingston 2016). This is important because the persistence of strong attitudes over time, which influence behaviour and receptivity to new information (Petty and Krosnick 1995), can prove challenging for conservation education and awareness efforts.

In our study, gender was also influential in people’s attitudes, albeit to a lesser degree relative to other covariates. Similar to an online survey on community attitudes towards flying foxes in Australia (Kung *et al.* 2015), male respondents were more likely to have a negative attitude towards flying foxes (Table 1A). Kellert and Berry’s (1987) seminal study of American adults throughout the United States also found gender one of the most important demographic factors influencing attitudes towards wildlife and conservation, as have subsequent studies (e.g., Rauwald and Moore 2002; Dougherty *et al.* 2003; Bjerke and Østdahl 2004; Miller and Jones 2006; Giam *et al.* 2015; Koziarski *et al.* 2016; Reid 2016). Our findings are consistent with this trend.

Less than one-third of respondents (30%) supported the idea that flying foxes should be conserved (Fig. 2), but those who did commonly cited the need for future generations to see and know flying foxes, with some expressing a moralistic concern that flying foxes are living creatures that have the right to exist. The majority of respondents (64%), however, stated that flying foxes are a problem for the village, likely the major reason for the desire to kill the bats, and the low support for their conservation. This corresponds to the high percentage of respondents (79%) who stated that they did not like flying foxes (Fig. 2), again the most common reasons being fruit raiding, noise, and mess/smell from faeces.

It is unclear to what extent people’s attitudes and perceptions may be biased by spouses or family members. Since we did not record the identity or address of our respondents, we acknowledge that our study did not manage to account for this possible bias in the data. The community in Juara is very tightly-knit however, with even separate households being very closely related through blood ties and marriage, and therefore we feel it would have been virtually impossible to tease this effect apart. Also, in Australia it has been found that people who live in closer proximity to flying fox roosts (<100 m) are more likely to have negative feelings about coexisting (Larsen *et al.* 2002). As we decided not to record the locations of respondents’ homes in order to maintain privacy, we were unable to assess whether this effect also exists in Juara.

### Implications and Recommendations for Conservation

#### Awareness and Outreach

Encouragingly, younger people are more likely to display positive attitudes towards flying foxes (Table 1A). Given the community’s low awareness of ecosystem services, it is clear that education and outreach efforts are needed (Walsh and Morton 2009), and our results suggest that younger audiences might be receptive to positive information about flying foxes. Education did not come up as an influencing factor in this study. Therefore, educational materials and awareness programs targeting schoolchildren, through cooperation and collaboration with teachers, could prove beneficial in spreading information about flying fox conservation, and improving attitudes towards bats in general. Successful examples of bat-centric community education programs (e.g., Kingston *et al.* 2006), including some for flying fox conservation (e.g., Trewhella *et al.* 2005), can be adapted for this purpose.

However, outreach and awareness should also include the older generation, particularly those who own fruit trees. Additionally, unsubstantiated beliefs regarding the medicinal properties of flying foxes need to be addressed. Our study revealed that cultural Chinese beliefs regarding the use of wildlife for medicinal purposes can also percolate through socio-cultural contact to influence perceptions of members of other ethnic groups. This appears to be an example of acculturation transmission, where influences are transmitted horizontally from one culture to another (Berry 1993). It is crucial to consider such cross-cultural exchanges when designing appropriate conservation messages and interventions in the multi-ethnic, multi-cultural context of a plural society such as Malaysia.

#### Mitigation of Fruit Raiding

The issue of fruit raiding needs to be addressed since it is frequently the reason why local communities, notably fruit tree owners, do not support fruit bat conservation (Aziz *et al.* 2016). In this study, older fruit tree owners who sell their fruit, and also experience raiding by flying foxes, are more likely to support the killing of flying foxes (Table 1B), indicating a slight economic dimension to the problem. Similar responses have been observed in Japan (Vincenot *et al.* 2015a), Mauritius (Florens 2015) and Costa Rica (Reid 2013). Williams-Guillén *et al.* (2016) point out that failure to address negative impacts of fruit bats not only compromises the conservation message, it may also result in actual bat fatalities, as seen in Mauritius (Florens 2016).

In Juara, the presence of tourists appears to act as a significant deterrent for fruit tree owners who may wish to eradicate flying foxes to protect fruit crops. However, if this perceived behavioural control (Kingston 2016) is removed, the frustrations of local people, whose beliefs and motivations are not conducive towards flying fox conservation, may jeopardise survival of flying foxes on Tioman. This situation illustrates the need for effective mitigation (Aziz *et al.* 2016).

#### Tourism Potential

The presence of tourists on Tioman likely generates social norm pressure (Kingston 2016) facilitating flying fox survival. Bat viewing is emerging as a growing tourist attraction that may help bat protection and conservation (Pennisi *et al.* 2004). The fact that flying foxes have not to date been highlighted as one of the island’s tourist attractions reveals the potential for added value. A large proportion of our respondents felt that flying foxes could be a tourist attraction (Fig. 4). Many, however, stated that Malaysian tourists dislike the bats and often complain about the noise and mess. Similar tensions exist in Australia (Bateman 2014; Drysdale 2014) and Cambodia (Chakrya 2008). It is thus essential to engage and collaborate with the host tourism community (Pennisi *et al.* 2004) in order to develop a consensus strategy that would yield benefits for local people.

Thus, the next step towards exploring the potential of bat tourism for Tioman is to conduct an awareness and perception survey targeting tourists to the island that would reveal whether there are obvious differences in attitudes and expectations amongst tourists that could guide appropriate conservation strategies to target both groups. Such a study could also help reveal whether tourists’ willingness-to-pay (e.g., through conservation levies) might be an incentive to elicit local community support for flying fox conservation, and how tourist expectations might be used to encourage more conservation-friendly behaviour among residents.

#### Disease Risk Management

Another interesting finding was the lack of strong concerns over disease risk (Fig. 4). This is consistent with other studies in Southeast Asia and Africa that found low perception of disease risk in people who were exposed to bats (Harrison *et al.* 2011; Robertson *et al.* 2011; Kamins *et al.* 2015), and is probably due to a lack of awareness. However, it is particularly surprising given the outbreak of Nipah virus in Peninsular Malaysia in 1998–99, where flying foxes were implicated as carriers (Wang 2009) and natural reservoir hosts for the virus, which first infected domestic pigs before jumping to humans (Looi and Chua 2007).

Zoonotic disease risk is real albeit low (Daszak *et al.* 2000; Mackenzie *et al.* 2003; Breed *et al.* 2006; Calisher *et al.* 2006; Luby 2013; Rahman *et al.* 2013; Schneeberger and Voigt 2016), and this issue needs to be considered and managed carefully in situations where humans coexist with synanthropic wildlife. High awareness of disease risk (Thiriet 2010; Snowdon 2016), compounded by sensationalised, alarmist and oftentimes misleading media reporting (e.g., Yang 2013), can be detrimental to both conservation and public health (Breed *et al.* 2006; Demma *et al.* 2009). This was demonstrated during the recent Ebola outbreak, despite research efforts consistently failing to prove that bats were the source of the virus (Tuttle 2015). Public health campaigns thus need to avoid inadvertently creating negative perceptions of bats – particularly as culling is not an effective means to prevent the spread of disease, and can actually be counter-productive by accelerating the viral prevalence. This underscores the need to adopt an interdisciplinary, ecosystem health approach that balances the wellbeing of humans, animals, and the environment (Daszak *et al.* 2004; Breed *et al.* 2006; One Health Initiative 2016).

## Conclusion

Despite no previous reports of conflict between humans and flying foxes on Tioman, concerns and negative attitudes amongst local people do exist – largely due to negative personal experiences living with the bats, and possibly lack of awareness on bat ecosystem services. These lead to perceptions that flying foxes are a useless nuisance requiring removal. The risk presented by zoonotic disease is an additional challenge that complicates the situation and can hamper efforts to promote more positive attitudes towards synanthropic flying foxes. This study shows that efforts to conserve flying foxes must be interdisciplinary in nature, employing a combination of different approaches. Although Kingston (2016) cautions against conservation approaches that emphasise utilitarian and materialist values, our results suggest that appealing to moralistic or altruistic values alone is insufficient. Despite some people acknowledging the intrinsic value of flying foxes as living beings, our results and observations suggest that the people on Tioman hold largely anthropocentric attitudes towards nature and wildlife – similar to that reported for Japan (Kellert 1991; Vincenot *et al.* 2015b). In the context of unpopular animals such as flying foxes, this is expressed principally through utilitarian and negativistic attitudes (see Kellert 1993). We believe that failure to respond with correspondingly appropriate conservation measures could ultimately result in a lack of local support for conservation. However, the potential for conservation success is promising. By using a combination of awareness, mitigation and tourism, it may yet be possible to effect positive changes in attitudes and behaviours, engage local communities positively, and produce a win-win conservation outcome.

## Electronic supplementary material

Below is the link to the electronic supplementary material.

ESM1 (DOCX 19.6 kb)

ESM2 (DOCX 15.1 kb)

ESM3 (DOCX 14.6 kb)
