# Supplementary material for: Coexistence and Conflict between the Island Flying fox (Pteropus hypomelanus) and Humans on Tioman Island, Peninsular Malaysia
Source: Hum Ecol Interdiscip J. 2017 Apr 24;45(3):377–89. doi: 10.1007/s10745-017-9905-6 (PMC5487769; doi:10.1007/s10745-017-9905-6)
Supplement: Supplementary file 1 — (DOCX 19.6 kb) [file 10745_2017_9905_MOESM1_ESM.docx]

SUPPLEMENTARY MATERIAL 1. List of questions used in questionnaire survey.

Date:

Enumerator:

Village (inc. GPS coordinates):

**1. Socio-demographics**

1. Gender: **□** Male **□** Female
2. How old are you?
3. Malaysian citizen? **□** Yes **□** No
4. Ethnic background:

**□** Chinese **□** Indian **□** Orang Asal **□** Orang Asli **□** Malay **□** Other **□** Mixed

1. What culture do you mainly practise?:

**□** Chinese **□** Indian **□** Orang Asal **□** Orang Asli **□** Malay **□** Other **□** Mixed **□** No specific culture

1. Religion: **□** Buddhist **□** Hindu **□** Islam **□** Christian **□** None **□** Other
2. Are you from here? **□** Yes **□** No

If no:

7a. Original village/hometown

7b. Current village/town

7c. How long have you lived here?

1. Formal education level:

**□** None

**□** Primary school

**□** Secondary School

**□** Diploma

**□** Degree

1. What is your occupation?
2. What is your main source of income?
3. On average, how much is your monthly income?

**□** None **□** <RM1000 **□** RM1000-2000 **□** RM2001-4000 **□** RM4001-7000 **□** >RM7000

1. Do you have any fruit trees? **□** Yes **□** No

If yes: 12a. Do you derive an income from your fruit trees? **□** Yes **□** No

If yes:

12b. How much income per month?

12c. Area size, type and number of fruit trees:

| Fruit tree type | Sell (*√ all)* | Own consumption  (*√ all)* | No. of trees |
| --- | --- | --- | --- |
| 1. |  |  |  |
| 2. |  |  |  |
| 3. |  |  |  |
| 4. |  |  |  |
| 5. |  |  |  |

**2. Knowledge and perceptions**

1. **(SHOW PHOTO)** Do you know what animal this is? **□** Yes **□** No
2. If yes, what is it called?
3. Do you know what flying foxes eat? **□** Yes **□** No
4. If yes, please describe.
5. Do flying foxes drink blood? **□** Yes **□** No **□** Don’t know

17a. How do you know?

1. Are flying foxes important for pollination? **□** Yes **□** No **□** Don’t know

18a. How do you know?

1. Can flying foxes be used as medicine? **□** Yes **□** No **□** Don’t know

19a. How do you know?

1. If yes, for what ailments?
2. Do flying foxes live in caves? □ Yes □ No □ Don’t know

21a. How do you know?

1. Do flying foxes disperse seeds? **□** Yes **□** No **□** Don’t know

22a. How do you know?

1. Do flying foxes carry viruses? **□** Yes **□** No **□** Don’t know

23a. How do you know?

1. If flying foxes disappeared (went extinct), what would happen?
2. Do you like flying foxes? □ Yes □ No □ Not sure

25a. Why?

1. Do you think flying foxes should be conserved? □ Yes □ No □ Not sure

26a. Why?

1. Do you think flying foxes are important for the environment? □ Yes □ No □ Not sure

27a. Why?

1. Do you think flying foxes bring benefits to humans? □ Yes □ No □ Not sure

28a. Why?

**3. Experiences**

1. Are there flying foxes living in this area? **□** Yes **□** No **□** Don’t know
2. Do flying foxes come to this area? **□** Yes **□** No **□** Don’t know
3. Did you ever see flying foxes in this area before? **□** Yes **□** No **□** Don’t know
4. If yes, when?

**□** Within this year **□** Within a year ago **□** Within 5 years ago **□** Within 10 years ago

**□** > 10 years ago

1. Do people come to hunt, eat or buy them? **□** Yes **□** No **□** Don’t know

If yes: 33a. What people, from where?

1. Do the flying foxes cause problems for the people here? **□** Yes **□** No **□** Don’t know

If yes: 34a. What problems?

34b. What have you tried to do to chase them away:

**□** Nothing **□** Shoot bats **□** Cut down tree **□** Put up nets **□** Light fires **□** Others:

1. Where do the flying foxes go when they fly out in the evening?

**4. Raiding (for fruit tree owners only)**

1. Do wild animals feed on your fruit trees?) **□** Yes **□** No

If yes: 36a. What animals eat your fruits:

Animal: ___________________ Tree: __________________________

36b. How often? (e.g. every night)

36c. When? (e.g. what time)

36d. During the year, which month/season do flying foxes visit your fruit trees the most?

1. Do flying foxes cause damage to your fruit trees? **□** Yes **□** No **□** Don’t know
2. If yes, what’s the average amount of your monthly financial loss?

**□** None **□** < RM100 **□** RM101-500 **□** RM501-1000 **□** > RM1000 **□** Don’t know

**5. Attitudes**

1. Do you think flying foxes should be killed? **□** Yes **□** No **□** Don’t know

39a. Why?

1. Do you think humans can catch diseases from flying foxes? **□** Yes **□** No **□** Don’t know

40a. Why?

1. Do you think flying foxes should be hunted for food and medicine? **□** Yes **□** No **□** Not sure

41a. Why?

1. Do you agree with a hunting ban? **□** Yes **□** No **□** Not sure

42a. Why?

1. Do you think flying foxes can be a tourist attraction? **□** Yes **□** No **□** Don’t know

43a. Why?

1. If flying foxes go extinct, would that be a good thing? **□** Yes **□** No **□** Don’t know

44a. Why?
