# Supplementary material for: Coexistence and Conflict between the Island Flying fox (Pteropus hypomelanus) and Humans on Tioman Island, Peninsular Malaysia
Source: Hum Ecol Interdiscip J. 2017 Apr 24;45(3):377–89. doi: 10.1007/s10745-017-9905-6 (PMC5487769; doi:10.1007/s10745-017-9905-6)
Supplement: Supplementary file 2 — (DOCX 15.1 kb) [file 10745_2017_9905_MOESM2_ESM.docx]

SUPPLEMENTARY MATERIAL 2. R code used for GLMMs.

##############################################################

## Import data

dat<-read.csv("XX.csv")

nrow(dat)

##Assign factors

dat$KIL<-as.factor(dat$KIL)

dat$INT<-as.factor(dat$ENU)

dat$GEN<-as.factor(dat$GEN)

dat$EDU<-as.factor(dat$EDU)

dat$LOC<-as.factor(dat$LOC)

dat$FRU<-as.factor(dat$FRU)

dat$INC<-as.factor(dat$INC)

dat$AGE<-scale(dat$AGE)

dat1<-data.frame(dat$KIL, dat$INT, dat$GEN, dat$LOC, dat$FRU, dat$AGE, dat$EDU, dat$INC, dat$ENU)

colnames(dat1)<-c("KIL","INT", "GEN", "LOC", "FRU", "AGE", "EDU", "INC", "ENU")

dat1 <- dat1[complete.cases(dat1),] ## complete cases

nrow(dat1)

##############################################################

## test correlations

library(psych)

### continuous vs. binary (use point bi-serial correlation = conventional pearson r)

cor(dat1$AGE, as.numeric(dat1$GEN)-1)

cor(dat1$AGE, as.numeric(dat1$LOC)-1)

cor(dat1$AGE, as.numeric(dat1$FRU)-1)

cor(dat1$AGE, as.numeric(dat1$EDU)-1)

cor(dat1$AGE, as.numeric(dat1$INC)-1)

### binary vs. binary (use phi coefficient)

phi(table(dat1$GEN, dat1$EDU))

phi(table(dat1$FRU, dat1$EDU))

phi(table(dat1$LOC, dat1$EDU))

phi(table(dat1$INC, dat1$EDU))

phi(table(dat1$GEN, dat1$FRU))

phi(table(dat1$GEN, dat1$LOC))

phi(table(dat1$GEN, dat1$INC))

phi(table(dat1$FRU, dat1$LOC))

phi(table(dat1$FRU, dat1$INC))

phi(table(dat1$LOC, dat1$INC))

## all coefficients below 0.5 (ok!)

##############################################################

## Load libraries

library(lme4)

library(MuMIn)

options(na.action = "na.fail")

GlobMod <- glmer(KIL ~ AGE + GEN + LOC + FRU + EDU + INCN+ (1|ENU), family=binomial(logit), dat1)

ModSetResult <- dredge(GlobMod, extra="R^2", rank="AICc")

AvgModSetResult <- model.avg(ModSetResult) ## variable importance

(AvgModSetResult$importance)

as.numeric((AvgModSetResult)$importance) ## for more decimal places

summary(AvgModSetResult)

ModSetResult ## Top 3 models are

## fit and get coefficients for top 3 models

m1 <- glmer(KIL ~ AGE + GEN + LOC + (1|ENU), family=binomial(logit), dat1) ## note that these models use ML estimation

m2 <- glmer(KIL ~ AGE + LOC + (1| ENU), family=binomial(logit), dat1)

m3 <- glmer(KIL ~ AGE + INCNY + LOC + (1| ENU), family=binomial(logit), dat1)

m0 <- glmer(KIL ~ (1| ENU), family=binomial(logit), dat1) ## null ## not used

## calculate marginal R^2 ## Nakagawa & Schielzeth 2013, download and paste code from https://www.dropbox.com/s/doy99n7s16c9mv6/rsquared.glmm?dl=0

R2m.m1

R2m.m2

R2m.m3
