# Supplementary material for: Coexistence and Conflict between the Island Flying fox (Pteropus hypomelanus) and Humans on Tioman Island, Peninsular Malaysia
Source: Hum Ecol Interdiscip J. 2017 Apr 24;45(3):377–89. doi: 10.1007/s10745-017-9905-6 (PMC5487769; doi:10.1007/s10745-017-9905-6)
Supplement: Supplementary file 3 — (DOCX 14.6 kb) [file 10745_2017_9905_MOESM3_ESM.docx]

SUPPLEMENTARY MATERIAL 3. Summary of respondent socio-demographic characteristics.

| **Characteristic** | **Category** | **No. (%) of respondents** |
| --- | --- | --- |
| Gender | Female  Male | 62 (52)  57 (48) |
| Age in years | <20  20-29  30-39  40-49  50-59  ≥60 | 3 (3)  14 (12)  31 (26)  30 (25)  24 (20)  16 (14) |
| Local to Juara? | Yes  No | 83 (70)  36 (30) |
| Education level | None  Primary  Secondary  Higher | 7 (6)  57 (49)  41 (34)  13 (11) |
| Monthly income in Malaysian ringgit (RM)* | None  <1000  1001-2000  2001-4000  4001-7000  >7000 | 19 (16)  47 (41)  34 (29)  10 (9)  4 (3)  2 (2) |
| Fruit tree ownership | Yes  No | 89 (75)  29 (25) |
| Sell fruits | Yes  No | 26 (32)  56 (68) |
| Experience raiding by flying foxes | Yes  No | 53 (60)  36 (40) |

*Respondent income categories were collapsed from six (Q11 in the questionnaire) to two (‘income’, ‘no income’) to facilitate regression analysis.
